# Genetic polymorphisms of CYP24A1 gene and cancer susceptibility: a meta-analysis including 40640 subjects

**DOI:** 10.1186/s12957-023-03156-w

**Published:** 2023-09-05

**Authors:** Yubin Wang, Ruiwen Wang, Shaofei Yuan, Xiaotang Liu

**Affiliations:** 1 Department of Radiotherapy, Ruian People’s Hospital, Ruian, 325200 Zhejiang China; 2 Gannan Medical University, Ganzhou, 341000 Jiangxi China; 3 Department of Oncology, Ruian People’s Hospital, Ruian, 325200 Zhejiang China; 4 Department of Urology, Ruian People’s Hospital, Ruian, 325200 Zhejiang China

**Keywords:** CYP24A1, Polymorphism, Cancer, Meta-analysis

## Abstract

**Background:**

Whether cytochrome P450 24A1 (CYP24A1) polymorphism is associated with cancer susceptibility, the individual study results are still controversial. Therefore, we performed a comprehensive study to identify the association of CYP24A1 polymorphisms (rs4809960, rs6068816, rs2296241, rs4809957, rs2762939) with cancer susceptibility.

**Methods:**

Electronic databases including Cochrane Library, PubMed, and Embase were systematically retrieved for relevant publications. Fixed or random-effect model was selected to calculate odds ratios (ORs) with their 95% confidence intervals (95%CI).

**Results:**

Eighteen published articles were identified. The results indicated that rs4809960 polymorphism was associated with a decreased cancer risk in Caucasian (TT vs. TC+CC: *P*=0.035; C vs. T: *P*=0.016) and Asian population (CC vs. TC+TT: OR *P*=0.044; TT vs. TC+CC: *P*=0.021; CC vs. TT: *P*=0.020; C vs. T: *P*=0.008) and breast cancer risk (TT vs. TC+CC: *P* = 0.007; TC vs. TT: *P*=0.004; C vs. T: *P*=0.033). A significant association was found between rs2296241 polymorphism and esophageal squamous cell carcinoma risk (AA vs. GG+AG: *P* = 0.023) and prostate cancer susceptibility (A vs. G: *P*=0.022). Furthermore, rs4809957 polymorphism was associated with prostate cancer susceptibility in Caucasian (GG vs. GA+AA: *P*=0.029; GA vs. GG: *P*=0.022) and breast cancer susceptibility (AA vs. GG+GA: *P*=0.012; AA vs. GG, *P*=0.010; A vs. G: *P*=0.024). Additionally, rs6068816 polymorphism significantly decreased the lung cancer (CC vs. CT+TT: *P* = 0.016; TT vs. CC: *P* = 0.044; CT vs. CC: *P* = 0.036; T vs. C: *P* = 0.016) and breast cancer risk (TT vs. CC+CT: *P* = 0.043; TT vs. CC: *P* = 0.039). No association was found for rs2762939 polymorphism with overall cancer risk. However, for rs2296241, rs4809957, and rs6068816 polymorphisms, there were no significant differences after the Bonferroni correction.

**Conclusion:**

The meta-analysis suggested that rs4809960 was associated with cancer risk and might be a genetic marker for predicting cancer risk. More large-scale and large-sample studies are necessary to further confirm these results.

**Supplementary Information:**

The online version contains supplementary material available at 10.1186/s12957-023-03156-w.

## Introduction

Cancer is a global public health problem, and incidence and mortality are rapidly growing worldwide. According to the data of the International Agency for Research on Cancer, GLOBOCAN 2020 investigation results showed 19.3 million new cancer cases and 10.0 million cancer deaths in 2020 [1]. In 2023, it is estimated that there will be 1,958,310 new cancer cases and 609,820 cancer-related deaths in the USA [2]. With the rapid growth and aging of the world population, the predominance of cancer is a leading cause of death. Current evidence suggests that factors, such as irregular lifestyles, smoking, alcohol intake, environmental factors, and genetic factors, are closely associated with the occurrence of cancer [1, 2]. Accumulative evidence has demonstrated that genetic factors may be associated with the etiology of cancer and the individual’s risk of cancer development, especially whole-genome association studies (GWAS) have identified various genes that may be involved in cancer development [3, 4].

Vitamin D, an essential fat-soluble vitamin, is mainly come from ultraviolet exposure and diet metabolism [5]. Meanwhile, it plays critical roles in cellular growth and anti-proliferative activities [6]. Clinical studies have indicated that vitamin D deficiency contributed to cancer risk, including prostate cancer, breast cancer, and thyroid carcinoma [7]. 25 hydroxy vitamin D (25(OH)D) is the main circulating form of vitamin D. In addition, 1,25 dihydroxy vitamin D (1,25(OH)2D3), an active form of vitamin D, which is associated with cell functions and gene expression. In the process of vitamin D metabolism, 25(OH)D and 1,25(OH)2D3 are converted to 24,25 dihydroxy vitamin D (24,25(OH)2D3) and 1,24,25 trihydroxy vitamin D (1,24,25(OH)3D3), respectively, which are degraded by 25-hydroxyvitamin D 24-hydrolase (encoded by CYP24A1 gene) [8]. Mutation of CYP24A1 may influence the metabolism of Vitamin D and anti-proliferative effects [9, 10].

Single-nucleotide polymorphisms (SNPs) are the most common form of variation in the human genome, which can alter the expression level or function of genes or their encoded products and thus determine the phenotype of the organism [11, 12]. Therefore, it is increasingly recognized that SNPs play a crucial role in the mechanisms of cancer [13]. Epidemiological studies have demonstrated that several common SNPs of CYP24A1 are involved in the concentration of circulating 25(OH)D [14]. To date, five common SNPs (rs4809960, rs6068816, rs2296241, rs4809957, rs2762939) were found to be associated with cancer risk, including esophageal squamous cell carcinoma prostate cancer, breast cancer, and lung cancer [5, 14, 15]. However, controversial results were reported and the association was not yet well established. Therefore, a comprehensive meta-analysis was performed to better explore the associations of CYP24A1 polymorphisms with cancer risk.

## Materials and methods

This study was performed under the guidelines of the Preferred Reporting Items for Systematic Reviews and Meta-analyses (PRISMA) and was registered in PROSPERO (CRD42023446451).

### Search strategy

The relevant paper was identified (published until Feb. 2023) through Embase, PubMed, and Cochrane Library using the following strategy: (CYP24A1 or rs2296241 or rs4809957 or rs2762939 or rs4809960 or rs6068816) and (polymorphism or SNP or variant or variation or mutation or genotype) and (cancer or carcinoma or tumor or neoplasm). In addition, other potential publications were also searched by scanning the reference list. The details of the search strategy can be found in Supplementary Table 1.

### Inclusion and exclusion criteria

Relevant studies were included according to the following criteria: (1) case-control studies, (2) evaluated the association between CYP24A1 polymorphism and cancer risk, (3) provided sufficient data to calculate the OR with 95%CI, and (4) control group conform to the Hardy–Weinberg equilibrium (HWE). The exclusion criteria were (1) review, abstract, comment, or letter; (2) duplication publications; and (3) relevant data not reported. In addition, for studies with repeat data, the study with the largest sample size was included. Each ethnicity was regarded as a separate study when different ethnicities were reported in a study.

### Data extraction and quality assessment

Two authors independently extracted relevant data from the included studies. The extraction parameters included the first author, publication year, country, ethnicity, sample size, cancer type, genotype, and allele distribution in cases and controls, and the *P* value of HWE in the control group, methodology quality of each study was assessed according to the Newcastle–Ottawa Scale (NOS).

### Statistical analyses

HWE was assessed by the chi-square test. The ORs and 95%CIs were calculated to evaluate the strength under allelic, recessive, dominant, homozygous, and heterozygous models. The *P* value of < 0.05 was considered as statistically significant. The chi-square test and *I*^2^ statistics were calculated to evaluate the heterogeneity across studies. If heterogeneity was found (*P*<0.10 or *I*^2^> 50%), the random-effect model was adopted. Otherwise, the fixed-effect model was adopted. Bonferroni correction was performed to adjust multiple-test *P* value [16]. Sensitivity analyses were performed to evaluate the stability of the results. Stratified analyses were performed by cancer type and ethnicity. Begg’s and Egger’s test was used to assess publication bias. Statistical analyses were completed using Stata 12.0 software (StataCorp, College Station, TX).

## Results

### Characteristics of the included studies

**Fig. 1:**
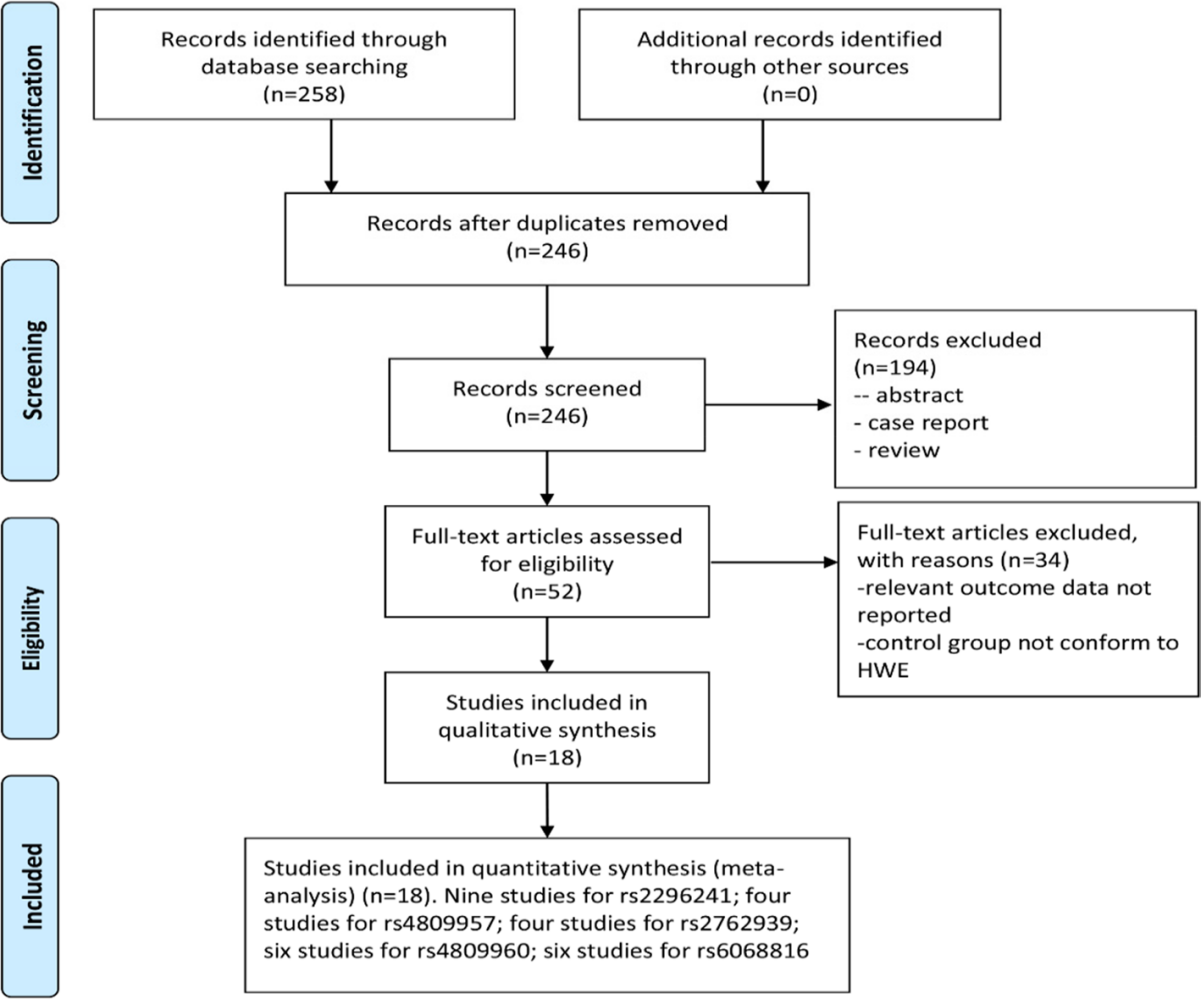
Flow chart of studies selection process

A total of 258 articles were retrieved in the initial search. Finally, a total of 18 articles [14, 15, 17–32] (19,017 cancer patients and 21,623 controls) were identified (Fig. 1). Among these 18 articles, nine publications about rs2296241 polymorphism, four publications focused on rs4809957 polymorphism, four on rs2762939 polymorphism, six on rs4809960 polymorphism, and six on rs6068816 polymorphism. In addition, four studies focused on prostate cancer, three on lung cancer, five on breast cancer, one on thyroid carcinoma, three on colorectal cancer, one on esophageal squamous cell carcinoma, and one on pancreas cancer. The characteristics of the included studies were described in Table 1.

**Table 1:** The main characteristics of the included studies

**First author**	**Year**	**Country**	**Ethnicity**	**Cancer type**	**Sample size**		**Case**			**Controls**			**HWE (control)**
					**Case**	**Control**	**AA**	**AB**	**BB**	**AA**	**AB**	**BB**	
**rs2296241**													
Holt a	2009	USA	Caucasian	Prostate cancer	692	705	166	356	170	151	371	183	0.147
Holt b	2009	USA	African	Prostate cancer	112	66	25	50	37	12	39	15	0.134
Wu	2017	China	Asian	Lung cancer	426	445	119	230	77	114	227	104	0.662
Holick	2007	UK	Caucasian	Prostate cancer	571	539	134	285	152	107	275	157	0.497
McCullough	2007	USA	Caucasian	Breast cancer	494	490	99	254	141	98	253	139	0.377
Penna-Martinez PTC	2012	Germany	Caucasian	Thyroid carcinoma	205	302	47	101	57	62	151	89	0.889
Penna-Martinez FTC	2012	Germany	Caucasian	Thyroid carcinoma	48	302	17	22	9	62	151	89	0.889
Yang	2017	China	Asian	ESCC	569	556	116	263	190	113	292	151	0.192
Anderson	2011	Canada	Caucasian	Breast cancer	1556	1630	330	777	449	371	791	468	0.294
Oh	2009	Korea	Asian	Prostate cancer	272	173	64(A%)	36(B%)		55.8(A%)	44.2(B%)		
Beuten	2011	USA	Caucasian	Prostate cancer	609	348	48.4(A%)	51.6(B%)		47(A%)	53(B%)		
Beuten	2011	USA	Caucasian	Prostate cancer	195	514	55.4(A%)	44.6(B%)		55.9(A%)	44.1(B%)		
Beuten	2011	USA	African	Prostate cancer	82	109	54.9(A%)	45.1(B%)		49.7(A%)	50.3(B%)		
**rs4809957**													
Anderson	2013	Canada	Caucasian	Pancreas cancer	627	1189	362	232	33	749	377	63	0.088
Zhuo	2018	China	Asian	Lung cancer	322	384	124	152	46	143	185	56	0.759
Gong	2017	China	Asian	Colorectal cancer	524	595	206	260	58	230	295	70	0.093
Wei	2019	China	Asian	Breast cancer	378	402	134	180	64	162	197	43	0.144
**rs2762939**													
Holt a	2009	USA	Caucasian	Prostate cancer	702	715	380	272	50	376	294	45	0.324
Holt b	2009	USA	African	Prostate cancer	114	67	29	48	37	13	32	22	0.824
Wu	2017	China	Asian	Lung cancer	426	445	160	192	74	156	220	69	0.553
Holick	2007	UK	Caucasian	Prostate cancer	568	539	319	212	37	300	196	43	0.173
Reimers	2015	USA	Caucasian	Breast cancer	921	970	514	348	59	560	353	57	0.889
**rs4809960**													
Holick	2007	UK	Caucasian	Prostate cancer	586	544	329	230	27	323	184	37	0.129
Holt a	2009	USA	Caucasian	Prostate cancer	697	693	432	220	45	387	260	46	0.794
Holt b	2009	USA	African	Prostate cancer	112	63	93	18	1	46	17	0	0.216
Reimers	2015	USA	Caucasian	Breast cancer	948	989	522	342	84	512	395	82	0.637
Clendenen	2015	Sweden	Caucasian	Breast cancer	733	1433	479	218	36	861	496	76	0.679
Sadeghi	2020	Iran	Caucasian	Colorectal cancer	220	243	119	95	6	105	119	19	0.062
Yi	2019	China	Asian	Colorectal cancer	787	800	415	311	61	468	290	42	0.736
**rs6068816**													
Holick	2007	UK	Caucasian	Prostate cancer	583	544	454	118	11	443	93	8	0.227
Holt a	2009	USA	Caucasian	Prostate Cancer	699	712	558	135	6	580	127	5	0.493
Reimers	2015	USA	Caucasian	Breast cancer	948	990	778	164	6	784	189	17	0.158
Clendenen	2015	Sweden	Caucasian	Breast cancer	733	1432	590	136	7	1149	264	19	0.389
Yi	2019	China	Asian	Colorectal cancer	787	800	342	354	91	362	348	90	0.645
Qu	2019	China	Asian	Lung cancer	345	351	160	155	30	131	178	42	0.116

*HWE* Hardy–Weinberg equilibrium, *ESCC* Esophageal squamous cell carcinoma, *a* Caucasian population, *b* African population, *A* wild type allele, *B* Mutated type allele

### Meta-analysis of rs4809960

Six publications [21, 26, 30–33] including seven studies (4509 cancer patients and 5210 controls) examined rs4809960 polymorphism. As shown in Table 2, no significant association between rs4809960 polymorphism and overall cancer susceptibility (Table 2). Subgroup analyses by ethnicity indicated that rs4809960 polymorphism was related to Caucasian population (TT vs. TC+CC: OR 1.18, 95%CI 1.01~1.37, *P*=0.035; C vs. T: OR 0.88, 95%CI 0.79~0.98, *P*=0.016) and Asian population (CC vs. TC+TT: OR 1.52, 95%CI 1.01~2.28, *P*=0.044; TT vs. TC+CC: OR 0.79, 95%CI 0.65~0.97, *P*=0.021; CC vs. TT: OR 1.52, 95%CI 1.08~2.48, *P*=0.020; C vs. T: OR 1.24, 95%CI 1.06~1.46, *P*=0.008). Subgroup analyses by cancer type revealed that rs4809960 polymorphism decreased breast cancer risk (TT vs. TC+CC: OR 1.19, 95%CI 1.05~1.36, *P* = 0.007; TC vs. TT: OR 0.82, 95%CI 0.72~0.94, *P*=0.004; C vs. T: OR 0.89, 95%CI 0.80~0.99, *P*=0.033). However, we only observed that rs4809960 polymorphism was significantly associated with the risk of breast cancer after Bonferroni correction.

**Table 2:** Summary of meta-analysis of association of rs4809960 polymorphism and cancer risk

**Comparison**		**Studies**	**Overall effect**				**Heterogeneity**	
			**OR (95% CI)**	***Z* score**	***p* value**	***p***_adjust_	***I***^**2**^** (%)**	***p* value**
CC vs. TC+TT	Overall	7	0.94 (0.71, 1.24)	0.43	0.667	1.000	51.6	0.054
	Caucasian	5	0.85 (0.65, 1.12)	1.14	0.255	1.000	43.9	0.129
	African	1	1.71 (0.07, 42.57)	0.33	0.744	1.000	-	-
	Asian	1	1.52 (1.01, 2.28)	2.01	0.044	0.308	-	-
	Prostate cancer	3	0.84 (0.60, 1.16)	1.08	0.280	1.000	0	0.480
	Breast cancer	2	1.01 (0.79, 1.30)	0.11	0.911	1.000	0	0.560
	Colorectal cancer	2	0.75 (0.17, 3.34)	0.37	0.708	1.000	88.4	0.003
TT vs. TC+CC	Overall	7	1.13 (0.94, 1.36)	1.30	0.192	1.000	74.3	0.001
	Caucasian	5	1.18 (1.01, 1.37)	2.10	0.035	0.245	57.4	0.052
	African	1	1.81 (0.86, 3.81)	1.56	0.118	0.826	-	-
	Asian	1	0.79 (0.65, 0.97)	2.31	0.021	0.147	-	-
	Prostate cancer	3	1.16 (0.81, 1.65)	0.82	0.415	1.000	72.9	0.025
	Breast cancer	2	1.19 (1.05, 1.36)	2.70	0.007	0.049	0	0.479
	Colorectal cancer	2	1.09 (0.56, 2.10)	0.25	0.804	1.000	89.9	0.002
CC vs. TT	Overall	7	0.90 (0.66, 1.23)	0.68	0.498	1.000	59.2	0.023
	Caucasian	5	0.81 (0.62, 1.06)	1.55	0.121	0.847	40.2	0.153
	African	1	1.49 (0.06, 37.34)	0.24	0.808	1.000	-	-
	Asian	1	1.64 (1.08, 2.48)	2.33	0.020	0.140	-	-
	Prostate cancer	3	0.81 (0.58, 1.13)	1.23	0.218	1.000	0	0.787
	Breast cancer	2	0.94 (0.73, 1.22)	0.45	0.650	1.000	0	0.538
	Colorectal cancer	2	0.71 (0.13, 4.05)	0.38	0.703	1.000	91.1	0.001
TC vs. TC+TT	Overall	7	0.89 (0.74, 1.07)	1.29	0.197	1.000	72.4	0.001
	Caucasian	5	0.86 (0.72, 1.02)	1.76	0.079	0.553	63.5	0.027
	African	1	0.52 (0.25, 1.11)	1.69	0.091	0.637	-	-
	Asian	1	1.21 (0.98, 1.49)	1.80	0.073	0.511	-	-
	Prostate cancer	3	0.86 (0.56, 1.32)	0.71	0.479	3.353	80.4	0.006
	Breast cancer	2	0.82 (0.72, 0.94)	2.87	0.004	0.028	0	0.602
	Colorectal cancer	2	0.95 (0.56, 1.60)	0.21	0.834	1.000	83.5	0.014
C vs. T	Overall	7	0.91 (0.79, 1.06)	1.24	0.217	1.000	73.3	0.001
	Caucasian	5	0.88 (0.79, 0.98)	2.40	0.016	0.112	43.1	0.135
	African	1	0.63 (0.32, 1.25)	1.32	0.185	1.000	-	-
	Asian	1	1.24 (1.06, 1.46)	2.67	0.008	0.056	-	-
	Prostate cancer	3	0.90 (0.74, 1.09)	1.07	0.284	1.000	42.3	0.177
	Breast cancer	2	0.89 (0.80, 0.99)	2.14	0.033	0.231	0	0.344
	Colorectal cancer	2	0.93 (0.51, 1.69)	0.25	0.804	1.000	92.5	<0.001

*OR* Odds ratio, *CI* Confidence interval

**Fig. 2:**
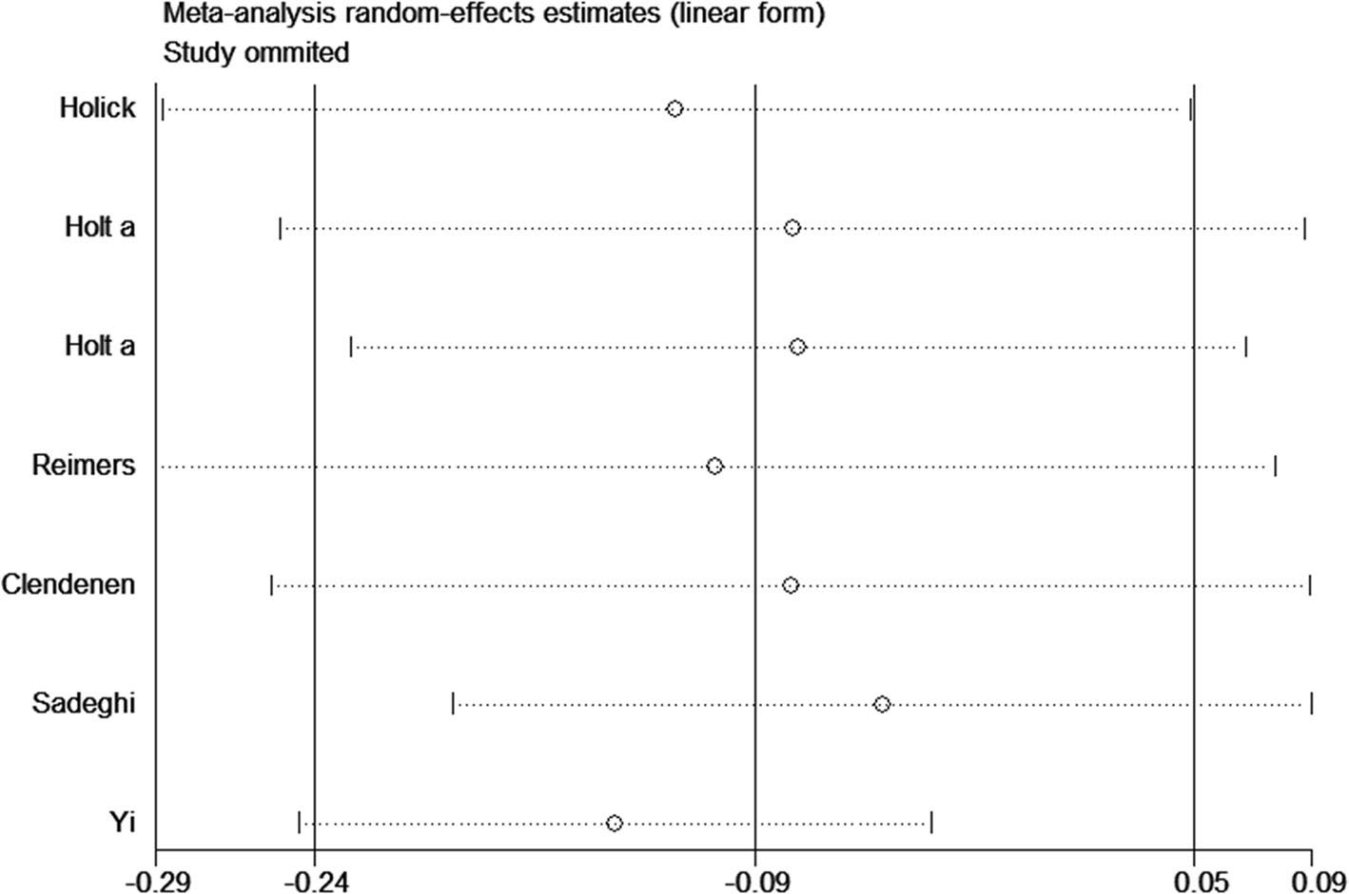
Sensitivity analysis for association between rs4809960 polymorphism and cancer risk (C vs. T)

Significant heterogeneity was found in all genetic models. Sensitivity analysis suggested that a significant association between rs4809960 polymorphism and overall cancer susceptibility was found (TT vs. TC+CC: OR 1.20, 95%CI 1.03~1.39, *P*=0.020, *I*^2^ = 53.1%; TC vs. TT: OR 0.84, 95%CI 0.70~0.99, *P*=0.043, *I*^2^ = 60.2%; C vs. T: OR 0.88, 95%CI 0.81~0.95, *P*=0.001, *I*^2^ = 37.2%) when after removed Yi et al. (Fig. 2). No visual publication bias was detected under the allelic genetic model. In addition, Egger’s test showed that there was no publication bias under the allelic genetic model (*P*=0.347).

### Meta-analysis of rs2296241

**Fig. 3:**
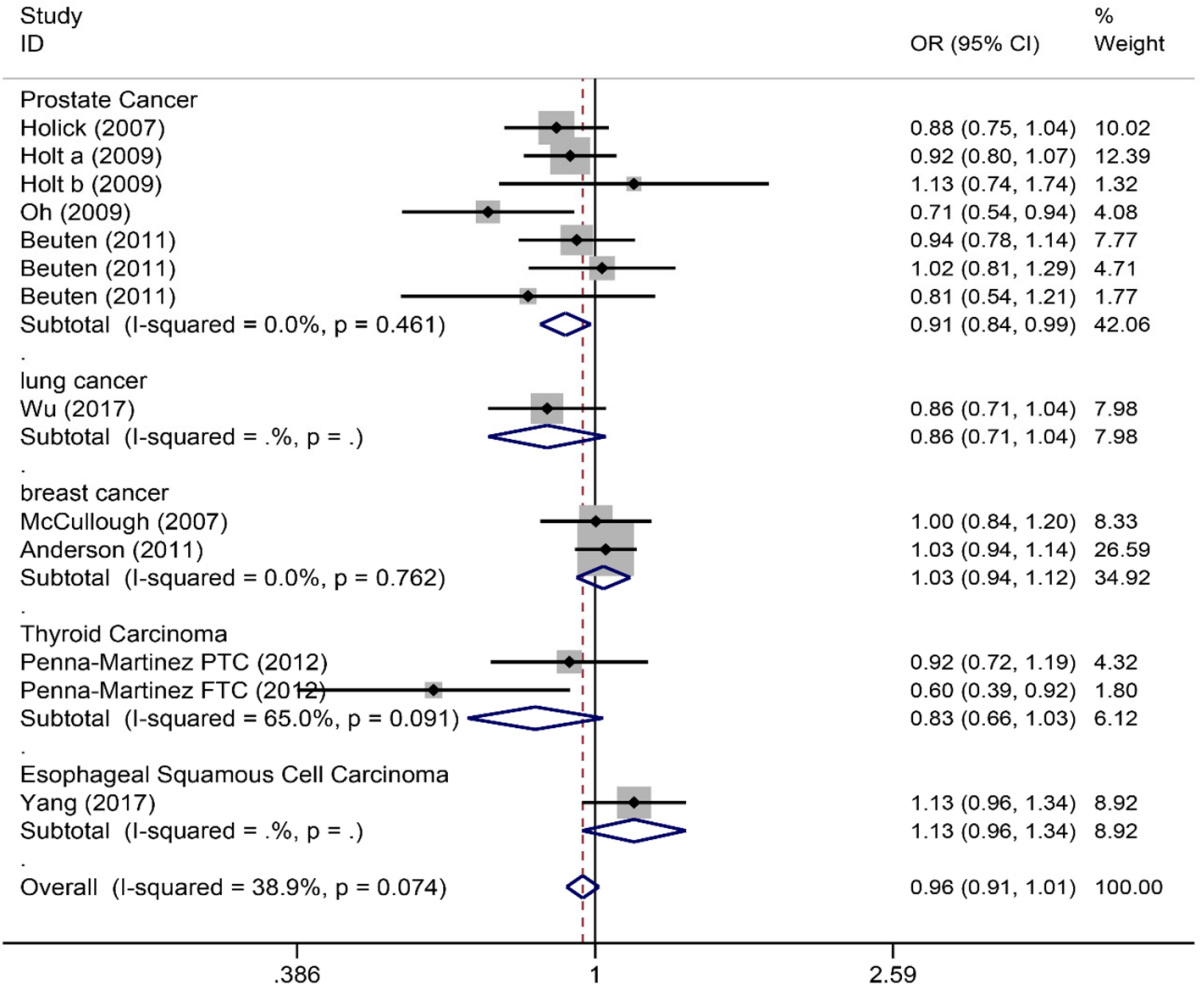
Forrest plot for association between rs2296241 polymorphism and cancer risk (A vs. G)

Nine publications [14, 15, 17, 19, 21–25] including 5831 cancer patients and 6179 controls were used to calculate pooled ORs and 95%CIs. As shown in Table 3, there was no significant association between rs2296241 polymorphism and overall cancer susceptibility in all genetic models. Subgroup analysis was performed according to ethnicity and cancer type. Stratification by ethnicity indicated that rs2296241 polymorphism was not related to ethnicity. In addition, subgroup analyses by cancer type revealed that rs2296241 polymorphism increased the risk of esophageal squamous cell carcinoma (AA vs. GG+AG: OR 1.34, 95%CI 1.04~1.74, *P* = 0.023) and decreased risk in prostate cancer (A vs. G: OR 0.91, 95%CI 0.84~0.99, *P*=0.022) (Fig. 3) (Table 3). However, these associations were no longer significant after the Bonferroni correction.

**Table 3:** Summary of meta-analysis of association of rs2296241 polymorphism and cancer risk

**Comparison**		**Studies**	**Overall effect**				**Heterogeneity**	
			**OR (95% CI)**	***Z* score**	***p* value**	***p***_adjust_	***I***^**2**^** (%)**	***p* value**
AA vs. GG+AG	Overall	9	0.99 (0.90, 1.08)	0.30	0.768	1.000	45.2	0.067
	Caucasian	6	0.96 (0.86, 1.06)	0.87	0.384	1.000	0	0.700
	African	1	1.68 (0.84, 3.37)	1.45	0.146	1.000	-	-
	Asian	2	1.06 (0.87, 1.30)	0.61	0.539	1.000	88.2	0.004
	Prostate cancer	3	0.94 (0.79, 1.12)	0.66	0.509	1.000	30.5	0.237
	Lung cancer	1	0.72 (0.52, 1.01)	1.92	0.055	0.385	-	-
	Breast cancer	2	1.01 (0.88, 1.15)	0.11	0.914	1.000	0	0.992
	Thyroid carcinoma	2	0.82 (0.58, 1.16)	1.12	0.261	1.000	26.6	0.243
	Esophageal squamous cell carcinoma	1	1.34 (1.04, 1.74)	2.27	0.023	0.161	-	-
GG vs. AA+AG	Overall	9	1.06 (0.96, 1.16)	1.13	0.260	1.000	18.1	0.282
	Caucasian	6	1.05 (0.94, 1.17)	0.89	0.371	1.000	45.7	0.101
	African	1	1.29 (0.60, 2.79)	0.66	0.512	1.000	-	-
	Asian	2	1.06 (0.86, 1.31)	0.56	0.579	1.000	0	0.592
	Prostate cancer	3	1.20 (1.00, 1.44)	1.93	0.054	0.378	0	0.923
	Lung cancer	1	1.13 (0.83, 1.52)	0.77	0.440	1.000	-	--
	Breast cancer	3	0.93 (0.80, 1.08)	0.92	0.355	1.000	0	0.607
	Thyroid carcinoma	2	1.37 (0.95, 1.96)	1.70	0.089	0.623	57.5	0.125
	Esophageal squamous cell carcinoma	1	1.00 (0.75, 1.34)	0.03	0.979	1.000	-	-
AA vs. GG	Overall	9	0.94 (0.84, 1.06)	1.00	0.317	1.000	38.5	0.112
	Caucasian	6	0.93 (0.82, 1.06)	1.07	0.285	1.000	40.1	0.138
	African	1	1.18 (0.48, 2.95)	0.36	0.717	1.000	0	-
	Asian	2	0.97 (0.75, 1.25)	0.23	0.820	1.000	76.9	0.037
	Prostate cancer	3	0.83 (0.67, 1.03)	1.67	0.096	0.672	0	0.682
	Lung cancer	1	0.71 (0.48, 1.05)	1.72	0.085	0.595	-	-
	Breast cancer	2	1.06 (0.89, 1.26)	0.67	0.501	1.000	0	0.735
	Thyroid carcinoma	2	0.68 (0.44, 1.05)	1.74	0.082	0.574	61.7	0.106
	Esophageal squamous cell carcinoma	1	1.23 (0.88, 1.71)	1.19	0.235	1.000	-	-
AG vs. GG	Overall	9	0.95 (0.85, 1.05)	1.05	0.292	1.000	1.7	0.420
	Caucasian	6	0.96 (0.86, 1.08)	0.64	0.521	1.000	25.6	0.243
	African	1	0.62 (0.27, 1.38)	1.18	0.238	1.000	-	-
	Asian	2	0.92 (0.74, 1.15)	0.72	0.469	1.000	0	0.653
	Prostate cancer	3	0.84 (0.69, 1.01)	1.81	0.070	0.490	0	0.718
	Lung cancer	1	0.97 (0.71, 1.33)	0.19	0.853	1.000	-	-
	Breast cancer	2	1.08 (0.92, 1.26)	0.94	0.346	1.000	0	0.581
	Thyroid carcinoma	2	0.76 (0.52, 1.12)	1.39	0.164	1.000	29.7	0.233
	Esophageal squamous cell carcinoma	1	0.88 (0.64, 1.19)	0.83	0.405	1.000	-	-
A vs. G	Overall	13	0.96 (0.91, 1.01)	1.49	0.137	0.959	38.9	0.074
	Caucasian	8	0.97 (0.91, 1.03)	1.13	0.257	1.000	18.2	0.286
	African	2	0.95 (0.70, 1.27)	0.37	0.714	1.000	20.1	0.263
	Asian	3	0.95 (0.84, 1.06)	0.96	0.339	1.000	79.4	0.008
	Prostate cancer	7	0.91 (0.84, 0.99)	2.29	0.022	0.154	0	0.461
	Lung cancer	1	0.86 (0.71, 1.04)	1.59	0.112	0.784	-	-
	Breast cancer	2	1.03 (0.94, 1.12)	0.61	0.543	1.000	0	0.762
	Thyroid carcinoma	2	0.83 (0.66, 1.03)	1.72	0.085	0.595	65.0	0.091
	Esophageal squamous cell carcinoma	1	1.13 (0.96, 1.34)	1.47	0.141	0.987	-	-

*OR* Odds ratio, *CI* Confidence interval

**Fig. 4:**
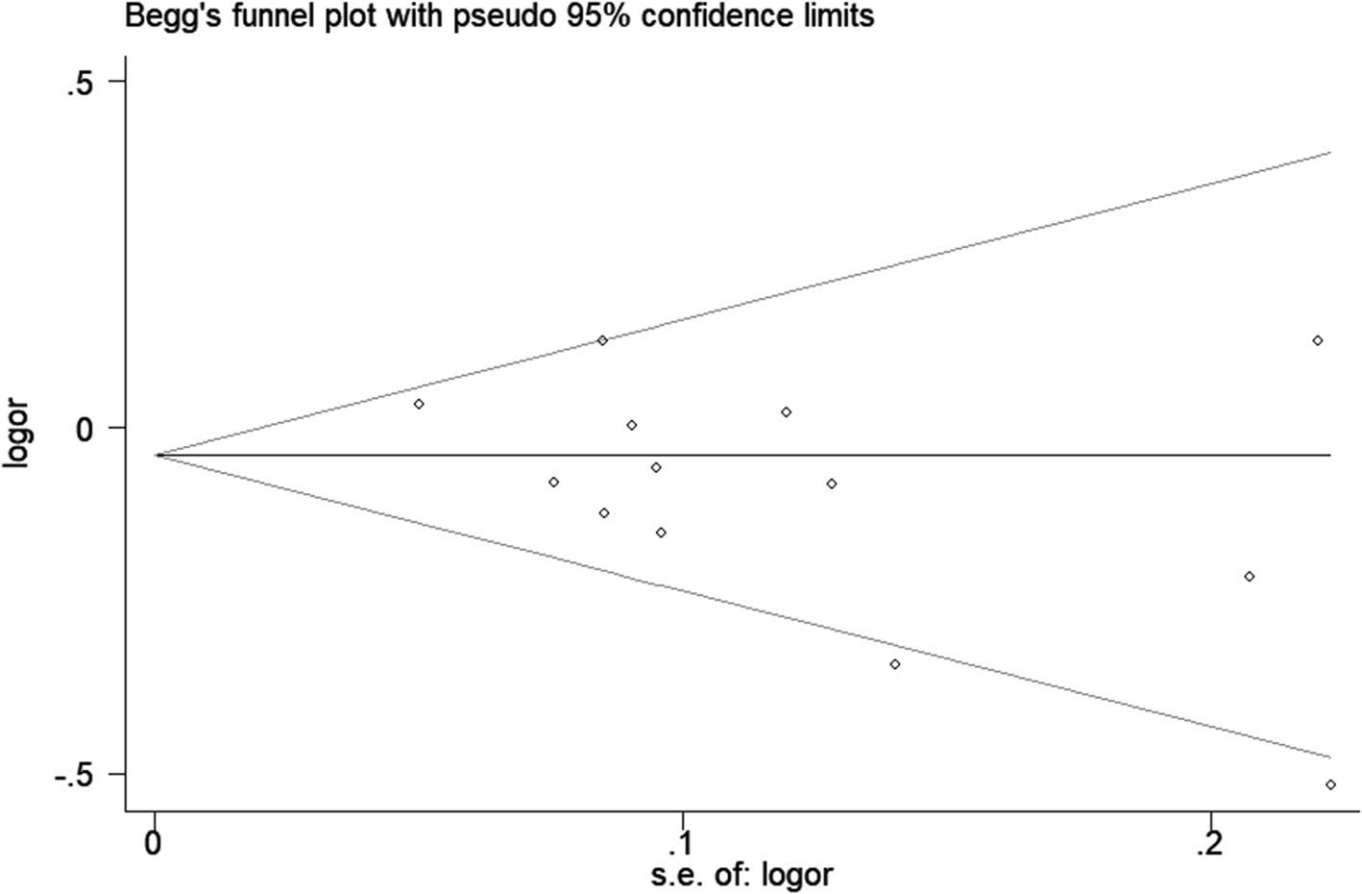
Begg’s funnel plot for association between rs2296241 polymorphism and cancer risk (A vs. G)

Significant heterogeneity was found under the recessive, homozygous, and allelic models. Sensitivity analysis showed that the initial result was not changed by removing each study respectively. No visual publication bias was detected under the allelic genetic model (Fig. 4). In addition, Egger’s test showed that there was no publication bias under the allelic genetic model (*P*=0.066).

### Meta-analysis of rs4809957, rs2762939 and rs6068816

**Fig. 5:**
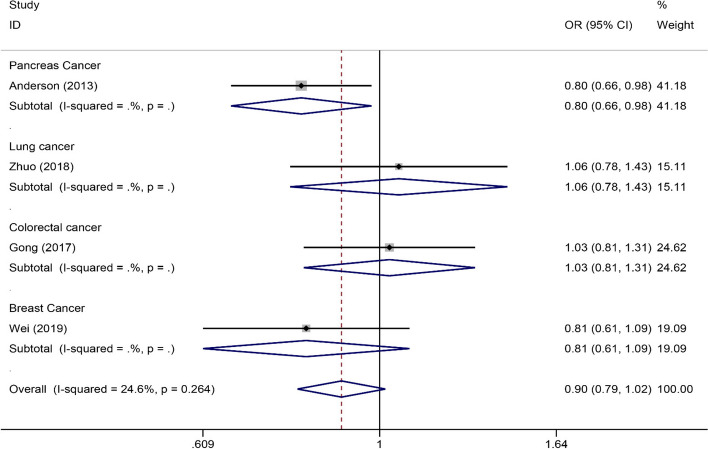
Forrest plot for association between rs4809957 polymorphism and cancer risk (GG vs. GA+AA)

**Fig. 6:**
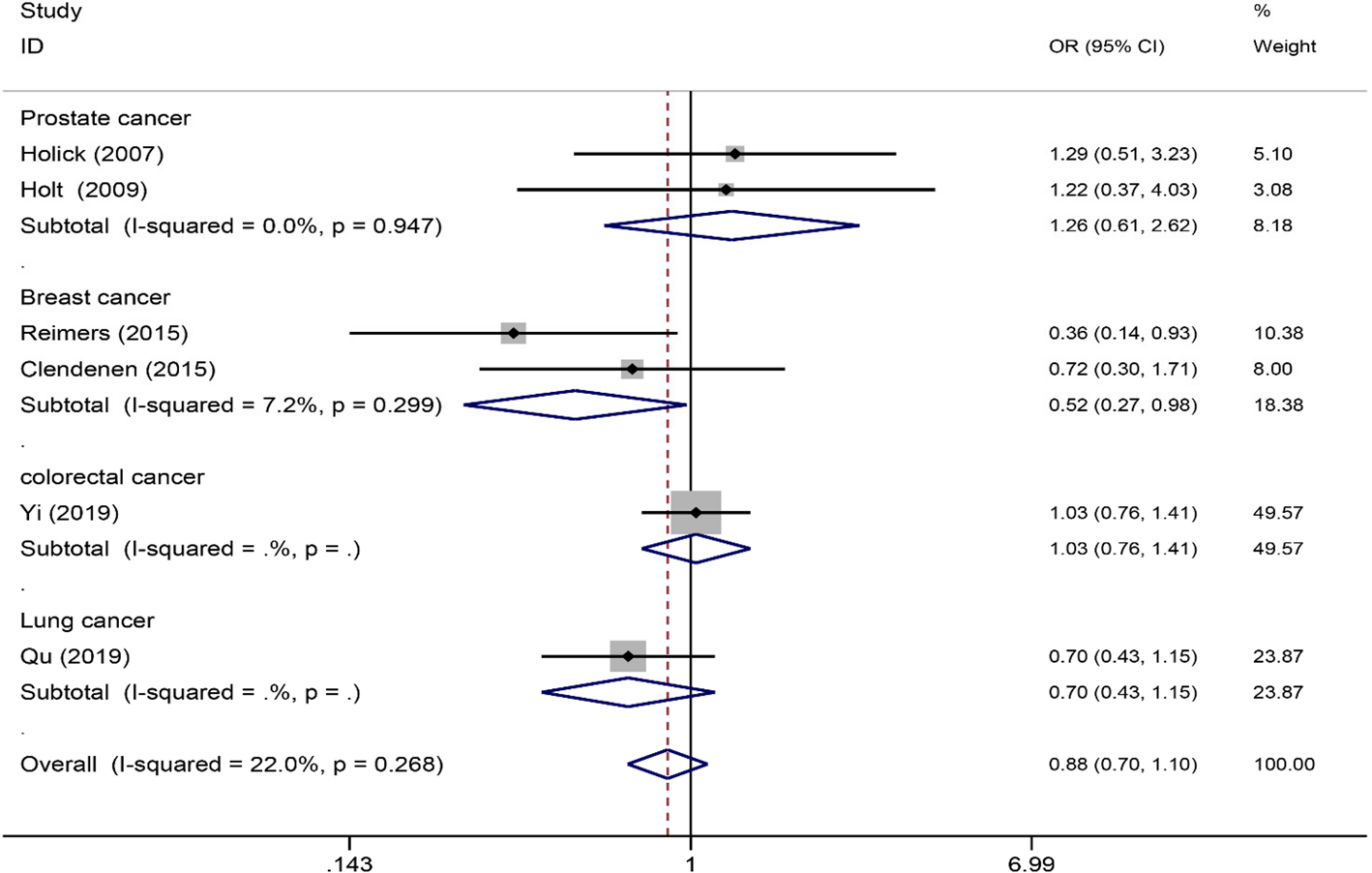
Forrest plot for association between rs6068816 polymorphism and cancer risk (TT vs. CC+CT)

Four publications [18, 20, 27, 28] (1851 cancer patients and 2570 controls) about rs4809957 polymorphism, four publications including five studies [15, 21, 22, 26] (2731 cancer patients and 2736 controls) about rs2762939 polymorphism and six publications [21, 26, 29–31, 33] (4095 cancer patients and 4829 controls) about rs6068816 polymorphism. As shown in Table 4, subgroup analyses revealed that rs4809957 polymorphism was significantly associated with Caucasian, especially pancreas cancer patients (GG vs. GA+AA: OR 0.80, 95%CI 0.66, 0.98, *P*=0.029; GA vs. GG: OR 1.27, 95%CI 1.04, 1.56, *P*=0.022). Furthermore, a significant association was found in breast cancer (AA vs. GG+GA: OR 1.70, 95%CI 1.22~2.58, *P*=0.012; AA vs. GG, OR 1.80, 95%CI 1.15~2.82, *P*=0.010; A vs. G: OR 1.27, 95%CI 1.03~1.55, *P*=0.024) (Fig. 5). In addition, there was no association between rs2762939 polymorphism with cancer risk (Table 5). For rs6068816, we found that rs6068816 polymorphism significantly decreased lung cancer (CC vs. CT+TT: OR 1.45, 95%CI 1.07~1.97, *P* = 0.016; TT vs. CC: OR 0.58, 95%CI 0.35~0.99, *P* = 0.044; CT vs. CC: OR 0.71, 95%CI 0.52~0.98, *P* = 0.036; T vs. C: OR 0.76, 95%CI 0.61~0.95, *P* = 0.016) and breast cancer risk (TT vs. CC+CT: OR 0.52, 95%CI 0.27~0.98, *P* = 0.043; TT vs. CC: OR 0.52, 95%CI 0.25~0.97, *P* = 0.039) (Fig. 6) (Table 6). However, these associations were no longer significant after the Bonferroni correction.

**Table 4:** Summary of meta-analysis of association of rs4809957 polymorphism and cancer risk

**Comparison**		**Studies**	**Overall effect**				**Heterogeneity**	
			**OR (95% CI)**	***Z* score**	***p* value**	***p***_adjust_	***I***^**2**^** (%)**	***p* value**
AA vs. GG+GA	Overall	4	1.11 (0.90, 1.36)	0.99	0.323	1.000	45.7	0.137
	Caucasian	1	0.99 (0.64, 1.53)	0.03	0.974	1.000	-	-
	Asian	3	1.14 (0.91, 1.44)	1.13	0.257	1.000	61.7	0.073
	Pancreas cancer	1	0.99 (0.64, 1.53)	0.03	0.974	1.000	-	-
	Lung cancer	1	0.98 (0.64, 1.49)	0.11	0.911	1.000	-	-
	Colorectal cancer	1	0.93 (0.64, 1.35)	0.36	0.715	1.000	-	-
	Breast cancer	1	1.70 (1.12, 2.58)	2.51	0.012	0.084	-	-
GG vs. GA+AA	Overall	4	0.90 (0.79, 1.02)	1.70	0.089	0.623	24.6	0.264
	Caucasian	1	0.80 (0.66, 0.98)	2.18	0.029	0.203	-	-
	Asian	3	0.97 (0.82, 1.13)	0.44	0.663	1.000	0	0.382
	Pancreas cancer	1	0.80 (0.66, 0.98)	2.18	0.029	0.203	-	-
	Lung cancer	1	1.06 (0.78, 1.43)	0.35	0.729	1.000	-	-
	Colorectal cancer	1	1.03 (0.81, 1.31)	0.23	0.822	1.000	-	-
	Breast cancer	1	0.81 (0.61, 1.09)	1.39	0.163	1.000	-	-
AA vs. GG	Overall	4	1.13 (0.91, 1.40)	1.11	0.265	1.000	47.4	0.127
	Caucasian	1	1.08 (0.70, 1.68)	0.36	0.720	1.000	-	-
	Asian	3	1.15 (0.89, 1.47)	1.08	0.282	1.000	64.7	0.059
	Pancreas cancer	1	1.08 (0.70, 1.68)	0.36	0.720	1.000	-	-
	Lung cancer	1	0.95 (0.60, 1.50)	0.23	0.817	1.000	-	-
	Colorectal cancer	1	0.93 (0.62, 1.37)	0.39	0.700	1.000	-	-
	Breast cancer	1	1.80 (1.15, 2.82)	2.56	0.010	0.070	-	-
GA vs. GG	Overall	4	1.10 (0.97, 1.26)	1.51	0.132	0.924	14.5	0.320
	Caucasian	1	1.27 (1.04, 1.56)	2.30	0.022	0.154	-	-
	Asian	3	1.01 (0.85, 1.19)	0.10	0.924	1.000	0	0.770
	Pancreas cancer	1	1.27 (1.04, 1.56)	2.30	0.022	0.154	-	-
	Lung cancer	1	0.95 (0.69, 1.31)	0.33	0.743	1.000	-	-
	Colorectal cancer	1	0.98 (0.77, 1.27)	0.13	0.900	1.000	-	-
	Breast cancer	1	1.10 (0.81, 1.50)	0.64	0.523	1.000	-	-
A vs. G	Overall	4	1.09 (0.99, 1.19)	1.73	0.084	0.588	45.7	0.137
	Caucasian	1	1.16 (0.89, 1.37)	1.80	0.071	0.497	-	-
	Asian	3	1.05 (0.94, 1.18)	0.87	0.387	1.000	55.9	0.104
	Pancreas cancer	1	1.16 (0.89, 1.37)	1.80	0.071	0.497	-	-
	Lung cancer	1	0.97 (0.78, 1.20)	0.30	0.763	1.000	-	-
	Colorectal cancer	1	0.97 (0.82, 1.15)	0.33	0.320	1.000	-	-
	Breast cancer	1	1.27 (1.03, 1.55)	2.25	0.024	0.168	-	-

*OR* Odds ratio, *CI* Confidence interval

**Table 5:** Summary of meta-analysis of association of rs2762939 polymorphism and cancer risk

**Comparison**		**Studies**	**Overall effect**				**Heterogeneity**	
			**OR (95% CI)**	***Z* score**	***p* value**	***p***_adjust_	***I***^**2**^** (%)**	***p* value**
CC vs. GG+GC	Overall	5	1.05 (0.87, 1.27)	0.51	0.609	1.000	0	0.774
	Caucasian	3	1.02 (0.80, 1.29)	0.17	0.866	1.000	0	0.479
	African	1	0.98 (0.52, 1.87)	0.05	0.958	1.000	-	-
	Asian	1	1.15 (0.80, 1.64)	0.74	0.458	1.000	-	-
	Prostate cancer	3	0.97 (0.74, 1.29)	0.18	0.857	1.000	0	0.538
	Lung cancer	1	1.15 (0.80, 1.64)	0.74	0.458	1.000	-	-
	Breast cancer	1	1.10 (0.75, 1.60)	0.48	0.631	1.000		
GG vs. CC+GC	Overall	4	1.02 (0.91, 1.13)	0.32	0.751	1.000	0	0.657
	Caucasian	3	0.99 (0.88, 1.12)	0.13	0.893	1.000	0	0.588
	African	1	1.42 (0.68, 2.96)	0.93	0.354	1.000	-	-
	Asian	1	1.11 (0.85, 1.47)	0.77	0.443	1.000	-	-
	Prostate cancer	3	1.06 (0.91, 1.23)	0.74	0.462	1.000	0	0.707
	Lung cancer	1	1.11 (0.85, 1.47)	0.77	0.443	1.000	-	-
	Breast cancer	1	0.92 (0.77, 1.11)	0.84	0.399	1.000		
CC vs. GG	Overall	4	1.01 (0.83, 1.23)	0.10	0.923	1.000	0	0.767
	Caucasian	3	1.02 (0.80, 1.0)	0.17	0.862	1.000	0	0.515
	African	1	0.75 (0.33, 1.75)	0.66	0.510	1.000	-	-
	Asian	1	1.05 (0.70, 1.55)	0.22	0.825	1.000	-	-
	Prostate cancer	3	0.93 (0.69, 1.25)	0.50	0.620	1.000	0	0.557
	Lung cancer	1	1.05 (0.70, 1.55)	0.22	0.825	1.000	-	-
	Breast cancer	1	1.13 (0.77, 1.65)	0.61	0.539	1.000		
GC vs. GG	Overall	4	0.97 (0.87, 1.09)	0.48	0.634	1.000	0	0.543
	Caucasian	2	1.01 (0.89, 1.14)	0.09	0.926	1.000	0	0.554
	African	1	0.67 (0.30, 1.49)	1.08	0.326	1.000	-	-
	Asian	1	0.85 (0.63, 1.14)	6.36	0.281	1.000	-	-
	Prostate cancer	3	0.94 (0.80, 1.11)	0.70	0.481	1.000	0	0.570
	Lung cancer	1	0.85 (0.63, 1.14)	1.08	0.281	1.000	-	-
	Breast cancer		1.07 (0.89, 1.30)	0.74	0.462	1.000	-	-
C vs. G	Overall	4	1.00 (0.92, 1.09)	0.02	0.984	1.000	0	0.834
	Caucasian	2	1.01 (0.92, 1.11)	0.18	0.860	1.000	0	0.589
	African	1	0.88 (0.57, 1.36)	0.59	0.554	1.000	-	-
	Asian	1	0.99 (0.81, 1.20)	0.14	0.892	1.000	-	-
	Prostate cancer	3	0.96 (0.85, 1.08)	0.66	0.512	1.000	0	0.884
	Lung cancer	1	0.99 (0.81, 1.20)	0.14	0.892	1.000	-	-
	Breast cancer	1	1.07 (0.92, 1.09)	0.87	0.382	1.000	-	-

*OR* Odds ratio, *CI* Confidence interval

**Table 6:** Summary of meta-analysis of association of rs6068816 polymorphism and cancer risk

**Comparison**		**Studies**	**Overall effect**				**Heterogeneity**	
			**OR (95% CI)**	***Z* score**	***p* value**	***p***_adjust_	***I***^**2**^** (%)**	***p* value**
TT vs. CC+CT	Overall	6	0.88 (0.70, 1.10)	1.13	0.259	1.000	22	0.268
	Caucasian	4	0.75 (0.47, 1.19)	1.23	0.219	1.000	30	0.232
	Asian	2	0.92 (0.71, 1.20)	0.59	0.553	1.000	40.8	0.194
	Prostate cancer	2	1.26 (0.61, 2.62)	0.63	0.527	1.000	0	0.947
	Breast cancer	2	0.52 (0.27, 0.98)	2.03	0.043	0.301	7.2	0.299
	Colorectal cancer	1	1.03 (0.76, 1.41)	0.20	0.845	1.000	-	-
	Lung cancer	1	0.70 (0.43, 1.15)	1.41	0.158	1.000	-	-
CC vs. CT+TT	Overall	6	1.03 (0.88, 1.20)	0.32	0.748	1.000	56.8	0.041
	Caucasian	4	0.99 (0.76, 1.13)	0.17	0.865	1.000	44.1	0.147
	Asian	2	1.45 (0.74, 1.77)	0.61	0.544	1.000	82.9	0.016
	Prostate cancer	2	0.85 (0.70, 1.04)	1.57	0.117	0.819	0	0.565
	Breast cancer	2	1.10 (0.94, 1.30)	1.18	0.236	1.000	6.9	0.300
	Colorectal cancer	1	0.93 (0.76, 1.13)	0.72	0.472	1.000	-	-
	Lung cancer	1	1.45 (1.07, 1.97)	2.42	0.016	0.112	-	-
TT vs. CC	Overall	6	0.86 (0.68, 1.09)	1.26	0.209	1.000	42.3	0.123
	Caucasian	4	0.75 (0.47, 1.20)	1.20	0.230	1.000	35.9	0.197
	Asian	2	0.90 (0.68, 1.19)	0.74	0.461	1.000	72.9	0.055
	Prostate cancer	2	1.31 (0.63, 2.71)	0.72	0.473	1.000	0	0.924
	Breast cancer	2	0.52 (0.27, 0.97)	2.06	0.039	0.273	13.6	0.282
	Colorectal cancer	1	1.07 (0.77, 1.48)	0.41	0.684	1.000	-	-
	Lung cancer	1	0.58 (0.35, 0.99)	2.01	0.044	0.308	-	-
CT vs. CC	Overall	6	1.00 (0.90, 1.10)	0.09	0.932	1.000	42.1	0.125
	Caucasian	4	1.02 (0.90, 1.16)	0.32	0.748	1.000	17.7	0.302
	Asian	2	0.95 (0.80, 1.13)	0.59	0.558	1.000	78	0.033
	Prostate cancer	2	1.16 (0.95, 1.42)	1.47	0.142	0.994	0	0.581
	Breast cancer	2	0.94 (0.80, 1.10)	0.78	0.435	1.000	0	0.409
	Colorectal cancer	1	1.08 (0.87, 1.33)	0.69	0.488	1.000	-	-
	Lung cancer	1	0.71 (0.52, 0.98)	2.10	0.036	0.252	-	-
T vs. C	Overall	6	0.97 (0.84, 1.11)	0.49	0.625	1.000	60.8	0.026
	Caucasian	4	1.00 (0.84, 1.19)	0.00	0.997	1.000	57.2	0.072
	Asian	2	0.90 (0.66, 1.24)	0.64	0.524	1.000	82.1	0.018
	Prostate cancer	2	1.16 (0.97, 1.39)	1.60	0.110	0.770	0	0.571
	Breast cancer	2	0.88 (0.74, 1.06)	1.36	0.173	1.000	32.1	0.225
	Colorectal cancer	1	1.05 (0.90, 1.21)	0.63	0.539	1.000	-	-
	Lung cancer	1	0.76 (0.61, 0.95)	2.42	0.016	0.112	-	-

*OR* Odds ratio, *CI* Confidence interval

Sensitivity analysis showed that removing each study respectively from the meta-analysis did not change the initial result. No publication bias was detected in the studies about rs4809957 and rs2762939 polymorphism meta-analysis.

## Discussion

CYP24A1, a member of the cytochrome P450 enzyme family, is located on the long arm of chromosome 20 (20q13.2). It is a key gene that converted 1,25(OH)2D3 to 1,24,25(OH)2D3 by 24-hydroxylation25-hydroxyvitamin D 24-hydrolase [34]. Albertson et al. [35] first identified the 20q13 gene amplification in breast cancer and identified the CYP24A1 gene as a candidate oncogene using array comparative genomic hybridization. CYP24A1 has been identified as a potential biomarker for cancer [36]. Numerous studies have suggested the expression level of the CYP24A1 was abnormally increased in several cancers, such as breast cancer, ovarian cancer, cervix carcinoma, lung cancer, and colon cancer [7, 37, 38]. Kong et al. [39] revealed that the rs6068816 and rs4809957 polymorphisms were associated with NSCLC risk. For breast cancer, Wei et al. [27] reported a significant association between the rs4809957 and breast cancer risk. Anderson et al. [18] revealed no significant correlation between rs4809957 with pancreas cancer. Among these publications reported the associations of CYP24A1 polymorphisms with cancer susceptibility, while the results remain controversial. The previous meta-analysis was performed by Zhu et al. [40], but they had not controlled the type I error rate through Bonferroni correction and had a smaller sample size. Therefore, the present meta-analysis aimed to re-evaluate the associations of CYP24A1 polymorphisms with cancer risk.

The present study indicated that there was no association between CYP24A1 polymorphisms (rs4809960, rs2296241, rs4809957, rs2762939, rs6068816) and overall cancer risk. For rs4809960 polymorphism, it was related to the Caucasian and Asian populations and decreased breast cancer risk. Moreover, our results suggested that rs2296241 polymorphism increased esophageal squamous cell carcinoma risk and decreased prostate cancer risk. For rs4809957 polymorphism, it was associated with pancreas cancer and breast cancer risk. In addition, we found that rs6068816 polymorphism significantly decreased lung cancer and breast cancer risk. However, rs4809960 polymorphism was associated with a decreased breast cancer risk after Bonferroni correction. A previous meta-analysis also reported CYP24A1 rs2296241 polymorphism was associated with prostate cancer risk [41]. Although our work found rs2296241 polymorphism was associated with an increased esophageal squamous cell carcinoma risk and decreased prostate cancer risk, these results could not withstand the Bonferroni correction.

The improvements of our meta-analysis are as follows: Firstly, more case-control studies about rs4809960, rs6068816, and rs2296241 polymorphism were included in the meta-analysis. Secondly, this is the first meta-analysis to assess the relationship between CYP24A1 (rs4809957, rs2762939) polymorphism and cancer risk. Thirdly, all included studies conform to the HWE, which may improve the reliability and stability of our study. In addition, all CYP24A1 polymorphisms were considered at the beginning. Ultimately, due to a lack of eligible articles and overlapping studies, our further evaluation of other CYP24A1 polymorphisms was limited. Therefore, in this meta-analysis, we only focused on five polymorphisms.

There are several limitations should be noted in the present study. First, the sample size of the included studies was relatively small, which might weaken the strength of the results. Second, the number of included studies in the subgroup analysis was also relatively small, which might lead to statistical bias. Third, not sufficient data to analyze whether environmental factors may influence the statistical result. Four, the association of CYP24A1 polymorphism with different types or stages, drinking, smoking, age, gender, exposure factors, or other risk factors was not considered in this study. Five, the patients of included studies mainly come from Caucasians. The African and Asian populations were relatively small.

In conclusion, this meta-analysis indicated that rs4809960 polymorphism was associated with a decreased breast cancer risk. No association between rs4809957, rs2296241, rs2762939, rs4809957 polymorphism, and overall cancer risk was found after Bonferroni correction. Considering the above limitations, more large-scale and large-sample studies are necessary to confirm these results.

### Supplementary Information

**Additional file 1: Supplementary Table 1.** Search strategy.

## Data Availability

The datasets used and/or analyzed during the current study are available from the corresponding author on reasonable request.

## Abbreviations

CYP24A1: Cytochrome P450 24A1
CI: Confidence interval
GWAS: Genome-wide association studies
HWE: Hardy-Weinberg equilibrium
NOS: Newcastle-Ottawa Scale
OR: Odds ratio
SNPs: Single-nucleotide polymorphisms
