# Supplementary material for: Genetic polymorphisms of CYP24A1 gene and cancer susceptibility: a meta-analysis including 40640 subjects
Source: World J Surg Oncol. 2023 Sep 5;21:279. doi: 10.1186/s12957-023-03156-w (PMC10478352; doi:10.1186/s12957-023-03156-w)
Supplement: Supplementary file 1 — Additional file 1: Supplementary Table 1. Search strategy. [file 12957_2023_3156_MOESM1_ESM.docx]

**Supplementary Information**

**Supplementary Table 1. Search strategy**

| Database | Pumed |
| --- | --- |
| Search | ("vitamin d3 24 hydroxylase"[Supplementary Concept] OR "vitamin d3 24 hydroxylase"[All Fields] OR "cyp24a1"[All Fields] OR "vitamin d3 24 hydroxylase"[MeSH Terms] OR ("vitamin"[All Fields] AND "d3"[All Fields] AND "24 hydroxylase"[All Fields]) OR "rs2296241"[All Fields] OR "rs4809957"[All Fields] OR "rs2762939"[All Fields] OR "rs4809960"[All Fields] OR "rs6068816"[All Fields]) AND ("polymorphic"[All Fields] OR "polymorphics"[All Fields] OR "polymorphism s"[All Fields] OR "polymorphism, genetic"[MeSH Terms] OR ("polymorphism"[All Fields] AND "genetic"[All Fields]) OR "genetic polymorphism"[All Fields] OR "polymorphism"[All Fields] OR "polymorphisms"[All Fields] OR ("socioaffect neurosci psychol"[Journal] OR "snp"[All Fields]) OR ("variant"[All Fields] OR "variant s"[All Fields] OR "variants"[All Fields]) OR ("variation"[All Fields] OR "variations"[All Fields]) OR ("mutate"[All Fields] OR "mutated"[All Fields] OR "mutates"[All Fields] OR "mutating"[All Fields] OR "mutation"[MeSH Terms] OR "mutation"[All Fields] OR "mutations"[All Fields] OR "mutation s"[All Fields] OR "mutational"[All Fields] OR "mutator"[All Fields] OR "mutators"[All Fields]) OR ("genotype"[MeSH Terms] OR "genotype"[All Fields] OR "genotypes"[All Fields] OR "genotypic"[All Fields] OR "genotype s"[All Fields] OR "genotyped"[All Fields] OR "genotyper"[All Fields] OR "genotypical"[All Fields] OR "genotypically"[All Fields] OR "genotyping"[All Fields] OR "genotypings"[All Fields] OR "genotypization"[All Fields])) AND ("cancer s"[All Fields] OR "cancerated"[All Fields] OR "canceration"[All Fields] OR "cancerization"[All Fields] OR "cancerized"[All Fields] OR "cancerous"[All Fields] OR "neoplasms"[MeSH Terms] OR "neoplasms"[All Fields] OR "cancer"[All Fields] OR "cancers"[All Fields] OR ("carcinoma"[MeSH Terms] OR "carcinoma"[All Fields] OR "carcinomas"[All Fields] OR "carcinoma s"[All Fields]) OR ("cysts"[MeSH Terms] OR "cysts"[All Fields] OR "cyst"[All Fields] OR "neurofibroma"[MeSH Terms] OR "neurofibroma"[All Fields] OR "neurofibromas"[All Fields] OR "tumor s"[All Fields] OR "tumoral"[All Fields] OR "tumorous"[All Fields] OR "tumour"[All Fields] OR "neoplasms"[MeSH Terms] OR "neoplasms"[All Fields] OR "tumor"[All Fields] OR "tumour s"[All Fields] OR "tumoural"[All Fields] OR "tumourous"[All Fields] OR "tumours"[All Fields] OR "tumors"[All Fields]) OR ("neoplasm s"[All Fields] OR "neoplasms"[MeSH Terms] OR "neoplasms"[All Fields] OR "neoplasm"[All Fields])) |
| Result | 114 |
| Database | Cochrane |
| Search | ((CYP24A1):ti,ab,kw OR (rs2296241):ti,ab,kw OR (rs4809957):ti,ab,kw OR ( rs2762939):ti,ab,kw OR ( rs4809960 ):ti,ab,kw OR ( rs6068816):ti,ab,kw OR MeSH descriptor: [CYP24A1] in all MeSH products)) AND ((polymorphism):ti,ab,kw OR (SNP):ti,ab,kw OR (variant):ti,ab,kw OR (variation):ti,ab,kw OR (mutation):ti,ab,kw OR (genotype):ti,ab,kw OR MeSH descriptor: [ polymorphism ] in all MeSH products)) AND  ((cancer):ti,ab,kw OR (carcinoma):ti,ab,kw OR (tumor):ti,ab,kw OR ( neoplasm ):ti,ab,kw OR MeSH descriptor: [cancer] in all MeSH products)) |
| Result | 10 |
| Database | Embase |
| Search | (CYP24A1 .ti,kw,hw OR rs2296241.ti,kw,hw  OR rs4809957.ti,kw,hw OR rs2762939.ti,kw,hw OR rs4809960.ti,kw,hw OR rs6068816.ti,kw,hw OR CYP24A1/exp) AND (polymorphism/exp OR polymorphism.ti,kw,hw OR SNP.ti,kw,hw OR variant.ti,kw,hw OR variation.ti,kw,hw OR mutation.ti,kw,hw OR genotype.ti,kw,hw) AND (cancer/exp OR cancer.ti,kw,hw OR carcinoma.ti,kw,hw OR tumor.ti,kw,hw OR neoplasm.ti,kw,hw) |
| Result | 134 |
